# A Potential Role of Coumestrol in Soybean Leaf Senescence and Its Interaction With Phytohormones

**DOI:** 10.3389/fpls.2021.756308

**Published:** 2021-11-25

**Authors:** Bong-Gyu Mun, Hyun-Ho Kim, Heung Joo Yuk, Adil Hussain, Gary John Loake, Byung-Wook Yun

**Affiliations:** ^1^School of Applied Biosciences, Kyungpook National University, Daegu, South Korea; ^2^Herbal Medicine Research Division, Korea Institute of Oriental Medicine (KIOM), Daejeon, South Korea; ^3^Department of Entomology, Abdul Wali Khan University, Mardan, Pakistan; ^4^Institute of Molecular Plant Sciences, University of Edinburgh, Edinburgh, United Kingdom

**Keywords:** coumestans, coumestrol, phytohormones, soybean, senescence (leaf)

## Abstract

Coumestrol is a natural organic compound synthesized in soy leaves and functions as a phytoalexin. The coumestrol levels in plants are reported to increase upon insect attack. This study investigates the correlation between coumestrol, senescence, and the effect of phytohormones on the coumestrol levels in soybean leaves. Our analysis involving high-performance liquid chromatography and 2-D gel electrophoresis indicated a significant difference in the biochemical composition of soybean leaves at various young and mature growth stages. Eight chemical compounds were specifically detected in young leaves (V1) only, whereas three different coumestans isotrifoliol, coumestrol, and phaseol were detected only in mature, yellow leaves of the R6 and R7 growth stage. MALDI-TOF-MS analysis was used to identify two proteins 3,9 -dihydroxypterocarpan 6A-monooxygenase (CYP93A1) and isoflavone reductase homolog 2 (IFR2) only in mature leaves, which are key components of the coumestrol biosynthetic pathway. This indicates that senescence in soybean is linked to the accumulation of coumestrol. Following the external application of coumestrol, the detached V1-stage young leaves turned yellow and showed an interesting development of roots at the base of the midrib. Additionally, the application of phytohormones, including SA, methyl jasmonate (MeJA), and ethephon alone and in various combinations induced yellowing within 5 days of the application with a concomitant significant increase in endogenous coumestrol accumulation. This was also accompanied by a significant increase in the expression of genes *CYP81E28* (Gm08G089500), *CYP81E22* (Gm16G149300), *GmIFS1*, and *GmIFS2*. These results indicate that various coumestans, especially coumestrol, accumulate during leaf maturity, or senescence in soybean.

## Introduction

Soybean [*Glycine max* (L.) Merr.] is an important crop. It is one of the most economical and valuable agricultural commodities because of its unique chemical composition. Due to its nutritional values, a diverse variety of soybean are consumed worldwide (Garro et al., 2004). It was also reported that the consumption of soybean can prevent many diseases and since it is rich in protein, phytoestrogens and contains low fat, it is useful for human consumption. The nutritional value of soybean is due to secondary metabolites that include various bioactive substances, including genistein, kaempferol, saponins, and coumestrol, which play critical roles in the human body (Burssens et al., 2011). Plant secondary metabolites are used as medicines, flavorings, and recreational drugs (Saxena et al., 2014). Besides, they also play vital roles and perform essential functions in plants (Seigler, 1998). The absence of secondary metabolites does not result in immediate death, long-term impairment of the organism’s survival, and fecundity, but plants use them as defensive chemicals to survive threats from various biotic and abiotic factors (Singh et al., 2012; Khan, 2014). Flavonoids belong to a significant group of secondary metabolites derived from the phenylpropanoid pathway. They are predominantly synthesized in legumes. Flavonoids are a type of naturally occurring secondary metabolites in higher plants, many of which act as phytoestrogens in mammals. One of the well-known potent phytoestrogens in soybean leaves is coumestrol, which functions as a phytoalexin when the plant is exposed to stressful conditions. Coumestrol also possesses anti-cancer and anti-obesity properties (Pham et al., 2019). It was reported that the coumestrol levels in soybean leaves increase during the mature stage (R7) (Ha et al., 2019). Additionally, research has shown that mature soybean leaves have α-glucosidase inhibitory activity. In a previous study, the most abundant polyphenols were isolated from soybean leaves and used to demonstrate that coumestrol was principally responsible for the potent α-glucosidase inhibition (Yuk et al., 2011b). As a useful substance for plant and human health, and its production is limited and influenced by environmental conditions. Thus, to discover a way to increase coumestrol levels in plants can be useful for industrial applications. As described above, the level of coumestrol in soybean leaves appears to increase as the plant ages. Additionally, an increase in coumestrol levels upon insect attack in some plants has been reported (Kain and Biggs, 1980). Senescence and insect damage are closely related to plant hormones, including ethylene (ET), jasmonic acid (JA), and salicylic acid (SA). This research investigates the correlation between coumestrol and senescence, and the interaction between coumestrol and phytohormones during soybean senescence. The changes in the chemical and protein composition of young and mature soybean leaves was analyzed with respect to aging and in response to phytohormone application using 2-dimensional gel electrophoresis (2-DE) and high-performance liquid chromatography (HPLC). The young leaves were selected at the vegetative growth stages V1, V3, and V5, whereas mature leaves were selected at the reproductive stages R1, R2, R4, R6, and R7.

## Materials and Methods

### Plant Materials and Treatment Conditions

Soybean plants (variety Daewon) were grown in pots inside a growth chamber at 30^*o*^C/14 h and 25^*o*^C/10 h, 60 – 70% relative humidity and at a sodium lamp light intensity of 1,000 μEm^2^/. The soybean leaves at the vegetative growth stages V1 (20 days), V3 (35 days), and V5 (56 days), and mature leaves at the reproductive stages R2 (77 days), R4 (98 days), R6 (112 days), and R7 (126 days) were used for the experiments. For the external application of coumestrol, soybean leaves at V1 growth stage were cut using sterilized scissors and immediately placed on a filter paper inside 125 × 125 × 20 mm square petri dishes (SPL, South Korea). Leaves were treated by supplying 5 ppm coumestrol [in 60% DMSO (dimethyl sulfoxide)] on the filter paper and incubated at 30/25°C (day/night) for 10 days. To avoid drying of samples, 5 ml of the solution was added after every 2 days (2, 4, 6, and 8 days). The samples were collected on the 1st, 3rd, 5th, and 10th day and stored at −80°C.

For phytohormone application, 20 days-old soybean plant leaves were cut and placed inside petri dishes as described above and the phytohormones SA (Sigma Aldrich), methyl jasmonate (MeJA) (Sigma Aldrich), and ethephon (Chaksak wang, farmhannong) were added at a concentration of 5 ppm alone and/or in different combinations. The samples were collected after 1, 3, 5, and 10 days after phytohormone applications. Control treatments were supplied with water.

### 2-Dimensional Gel Electrophoresis Analysis and Protein Identification

Total protein from soybean leaves was extracted using the Mg/NP-40 buffer, which was fractionated with PEG 4000, following the method described by Kim et al. (2001, 2003). Each sample (150 μg) was mixed with isoelectric focusing (IEF) sample buffer and loaded onto an 18-cm IEF gel. The second-dimension separation was conducted on S sodium dodecyl sulfate poly acrylamide gel electrophoresis (SDS-PAGE) using 12% polyacrylamide gels. 2-DE gels were silver-stained (Kavran and Leahy, 2014), scanned (PowerLook III, UMAX) and exported as TIFF files from the scanner, after which gel spots were detected using ImageMaster 2D Platinum software (Amersham Biosciences). Differentially expressed protein spots were identified using MALDI-TOF-MS. Gel spots digested with trypsin were analyzed using a Voyager-DE STR (matrix-assisted laser desorption ionization time-of-flight) and MALDI-TOF mass spectrometer (PerSeptive Biosystems). Briefly, individual protein spots were isolated and remelted with digestion mixtures [93:5:2 water, acetonitrile, and trifluoroacetic acid (TFA)]. Then, the samples were sonicated for 5 min and centrifuged; 2-μl sample was added to 2-μl peptide sample solution (the matrix solution, the nitrocellulose solution, and isopropanol were mixed 100: 50: 50 (Kim et al., 2004), and 1 μl of this was placed on the MALDI plate and left for 5 min, after which the samples were washed with 0.1% v/v TFA. Des-Arg1-bradykinin (m/z 904.4681) and angiotensin 1 (m/z 1296.6853) were used as internal standards for calibration. For data analysis, the PerSeptive-Grams software was utilized. Database searches for protein identification were performed using Protein Prospector.^1^

### Detection of Secondary Metabolites *via* UPLC

For secondary metabolites analysis, 1 g of soybean leaves were extracted with 20 mL of 70% ethanol for 12 h and centrifuged at 3,000 rpm for 5 min. One milliliter of supernatant was filtered through a 0.2 μm PTFE filter and subjected to metabolite analysis. Chromatographic separation was performed using a UPLC system (Waters Corp., Milford, MA, United States) equipped with a binary solvent delivery system and a UV detector with absorbance at 254 nm. Aliquots (2.0 μl) of each sample were then injected into a BEH C18 column (2.1 × 100 mm, 1.7 μm) at a flow rate of 0.4 ml/min and eluted using a chromatographic gradient of two mobile phases (A: water containing 0.1% formic acid; B: acetonitrile containing 0.1% formic acid). A linear gradient program was followed: 0 min, 10% B; 0–7 min, 10–15% B; 7–11 min, 15–30% B; 11–16 min, 30–50% B; 16–17 min, 80–100% B; 17–18 min, 100% B, 18.3–20 min, back to 10% B.

### Coumestrol Levels and Hormone Analysis by High-Performance Liquid Chromatography

The measurements of coumestrol and plant hormones were performed as described by Pan et al. (2010) and Ng et al. (2016) with minor modifications using flight (LC-ESI-QTOF) tandem mass spectrometry. Soybean leaves (1 g) were extracted with 80% MeOH aq. (10 mL) and shaken for 24 h at room temperature. The filtered solution was analyzed using Liquid Chromatography Electrospray-Ionization Quadrupole Time-of-flight (LC-ESI-QTOF) Tandem Mass Spectrometry. The hormones were analyzed using the method described by Pan et al. (2010). Extraction solutions I, II, and internal standards were prepared. Extraction solvent 1 [2-propanol/H2O/0.5% TFA (Trifluoroacetic acid)]. Extraction solution II; dichloromethane. A working solution of internal standards was prepared by diluting the combined stocks with methanol. Frozen plant tissues (100 mg) were ground and transferred into an E-tube, then 1-ml extraction solution I was added and shaken at 4°C for 30 min. After that, 1-ml extraction solution II was added and kept at 4°C for 30 min, after which it was centrifuged at a revolution speed of 13,000 rpm at 4°C for 5 min. The aqueous phase was transferred to a new tube, and 600-μl isopropanol was added to the sample and mixed gently. The samples were incubated at room temperature for 10 min and centrifuged at 13,000 rpm for 10 min at 4°C. The supernatant was discarded. The pellet was washed with 1-ml 75% EtOH. The samples were air-dried, and the pellet was resuspended in 50-μl nuclease-free water to dissolve the pellet.

Soybean leaf metabolites were identified using MS (accurate mass in negative mode) or MS/MS spectra (fragmentation pattern), UV/Vis spectra, and in-house library comparison as described by Yuk et al. (2011b). Calculations were based on the area of HPLC analysis. Values, reported as relative contents vs. the amount of individual coumestrol at R4 stage, are the mean measurements carried out on three independent samples analyzed three times.

For quantification of coumestrol, we used external standard measurement. Four different concentrations (10, 100, 1,000, and 10,000 ppm) of coumestrol (27,885, Sigma-Aldrich, 95%, HPLC) were used and generated standard curve. The value from the standard were then used to calculate concentration of coumestrol in samples. All the steps were carried out using a preinstalled software (MassLynx 4.1).

### Quantitative Real-Time Polymerase Chain Reaction Analysis

The total RNA was extracted using Trizol TRI Reagent Solution (ThermoScientific) according to the manufacturer’s standard protocol, cDNA was synthesized according to the manufacturer’s instructions from the total RNA using the reverse transcription BioFact^TM^ RT Kit. The real-time polymerase chain reaction was conducted using an Eco^TM^ real-time PCR system (Illumina) using 2× Real-Time PCR Master Mix, including SYBR Green I (Biofact) and 10 pmol of each primer, processed in a two-step PCR program. All analyses were conducted in triplicate, and gene expression data were analyzed using ECO Study program (Illumina). Relative expression levels were determined by testing treated and control plants, and normalized to *GmActin*.

## Results

### Linkage of Soybean Leaf Maturity to the Accumulation of Coumestans

**FIGURE 1:**
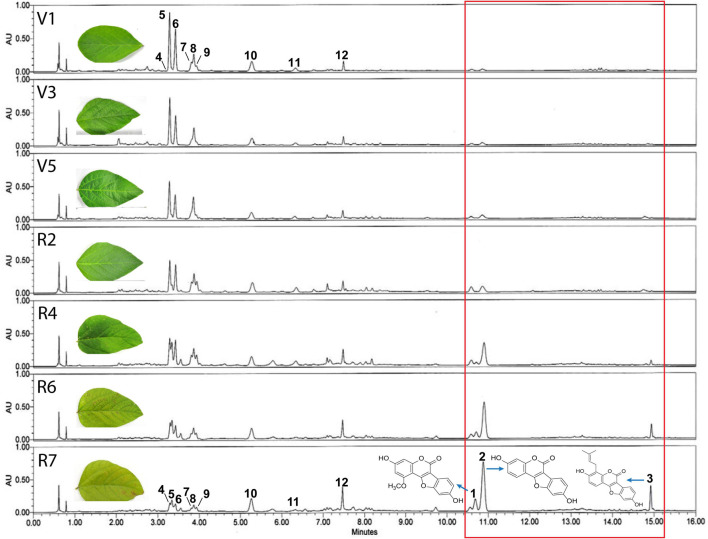
Representative UPLC-PDA (λ_*max*_) chromatograms. Representative chromatograms of compounds detected in soybean leaf extracts at different growth stages (vegetative: V1, V3, V5, and reproductive R4, R6, and R7). Results showed significant differences in the chemical composition of the leaves at various growth stages, as reflected by detecting diverse chemical compounds in the leaves collected at various growth stages. Eight chemical compounds (peaks 5–12) detected were found to have significantly higher accumulation in the fresh leaves (V1) compared with mature leaves (R7). Furthermore, three major chemical groups present were detected only in mature yellow leaves of the R7 growth stage (red box, peaks 1, 2, and 3).

Soybean leaves are known to turn yellow upon maturity as the development and maturity of pods begin. Cutting the supply of food to several yellowing leaves helps divert and accumulate nutrients in the pods for future generation. Leaf yellowing and plant maturity also relate to gradual variations in the day and night length, which significantly affects soybean senescence. Previously, it was reported that mature soybean leaves with yellow color inhibit the α-glucosidase activity due to high coumestrol levels (Yuk et al., 2011a). The color of soybean leaves begins to turn yellow from R2 onward, and completely yellow leaves develop at the mature or senescent R7 stage (Supplementary Figure 1A). The color change or senescence is accompanied by a concomitant loss of the total chlorophyll content. Our results indicated that significant increase in the chlorophyl content occurs up to V5 and then starts to reduce, with the least chlorophyl content at the R7 stage (Supplementary Figure 1B). To verify the correlation between leaf color changes or senescence and leaf metabolites, soybean leaves at different growth stages were collected after sowing i.e., 20 days (V1), 35 days (V3), 56 days (V5), 77 days (R2), 98 days (R4), 112 days (R6), and 126 (R7) days after sowing and the metabolite profile of the leaves were analyzed at the above-mentioned growth stages. HPLC analysis showed diverse and significant differences in the chemical composition of the leaves at the various growth stages, as reflected by detecting diverse chemical compounds in the leaves collected at various growth stages (Figure 1). More specifically, we identified three major chemical groups that were significantly abundant in mature, yellow leaves of the R7 growth stage (Figure 1, red box, peaks 1, 2, and 3). Although these three groups were also detected in the earlier growth stages, their quantities were significantly lower, as reflected by the smaller peaks in the chromatogram). Additionally, at least eight other chemical compounds (Figure 1, peaks 5–12) detected had a significant quantitative difference between fresh and mature leaves as significantly lower quantities were detected in mature leaves (R7). Among these, compounds representing peaks 5–9 were identified as flavonoid glycosides, while those representing peaks four, ten, eleven, and twelve were identified as glycosyl-oxy-isoflavones. These results are parallel with the previous work of Yuk et al. (2016). The chemical compounds representing peaks one, two, and three at the R7 stage were identified as coumestans. These three chemical compounds were identified as Isotrifoliol (peak 1), Coumestrol (peak 2), and Phaseol (peak 3). This indicates that coumestans play a key role in soybean leaf maturity. Additionally, among these three compounds, coumestrol (peak 2) was the most abundant chemical compound at the R7 stage.

**FIGURE 2:**
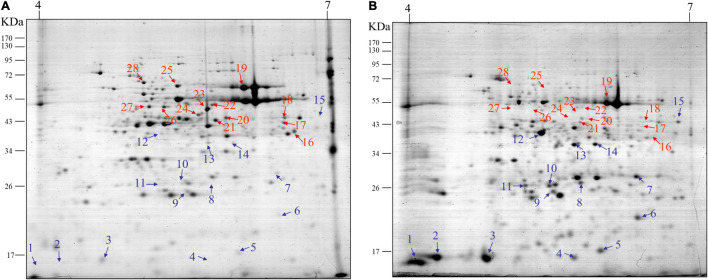
Results of 2-DE analysis of proteins extracted from soybean leaves. Representative 2-DE gel image showing proteins extracted from soybean leaves at the V1 stage **(A)** and R7 stage **(B)**. At least 28 proteins showed significant differences between the two gels. Among these, 15 proteins showed a significantly high accumulation (blue arrows) whereas, 13 showed significant reduction (red arrows) at the R7 stage **(B)** compared to the V1 stage **(A)**.

Next, we conducted 2-D gel electrophoresis (2-DE) to separate various proteins extracted from leaves at two growth stages V1 and R7. Since the 2-DE separates proteins based on two properties in two dimensions, it was better suited to separate diverse proteins expressed at specific growth stages. The results indicated the detection of highly diverse proteins spread across both gels. Additionally, significant differences were observed in terms of the number, intensity, and positions of proteins/spots on the two gels, corresponding to the two growth stages, V1 and R7 (Figures 2A,B, respectively). For further detailed analysis, 28 different proteins were selected (represented by 28 spots on the gel). Among these, 15 proteins showed a significantly high accumulation (Figure 2, blue arrows) whereas 13 showed significant reduction (Figure 2, red arrows) in the gel corresponding to the R7 stage compared to the gel corresponding to the V1 stage. These proteins were further analyzed using MALDI-TOF-MS. A total of 19 proteins/spots were successfully identified, including 12 proteins that showed higher accumulation and seven proteins that showed a reduction at the R7 stage compared to V1. A list of the identified proteins is given in Supplementary Table 1. Enzymes, including malate synthase, alanine aminotransferase, and glutamate semialdehyde aminomutase, were identified among the proteins with lower accumulation in mature leaves at the R7 stage. However, different proteins involved in secondary metabolite biosynthesis were identified among the proteins with significantly higher accumulation in mature leaves.

### Role of Coumestrol in Leaf Color Change and Senescence

**FIGURE 3:**
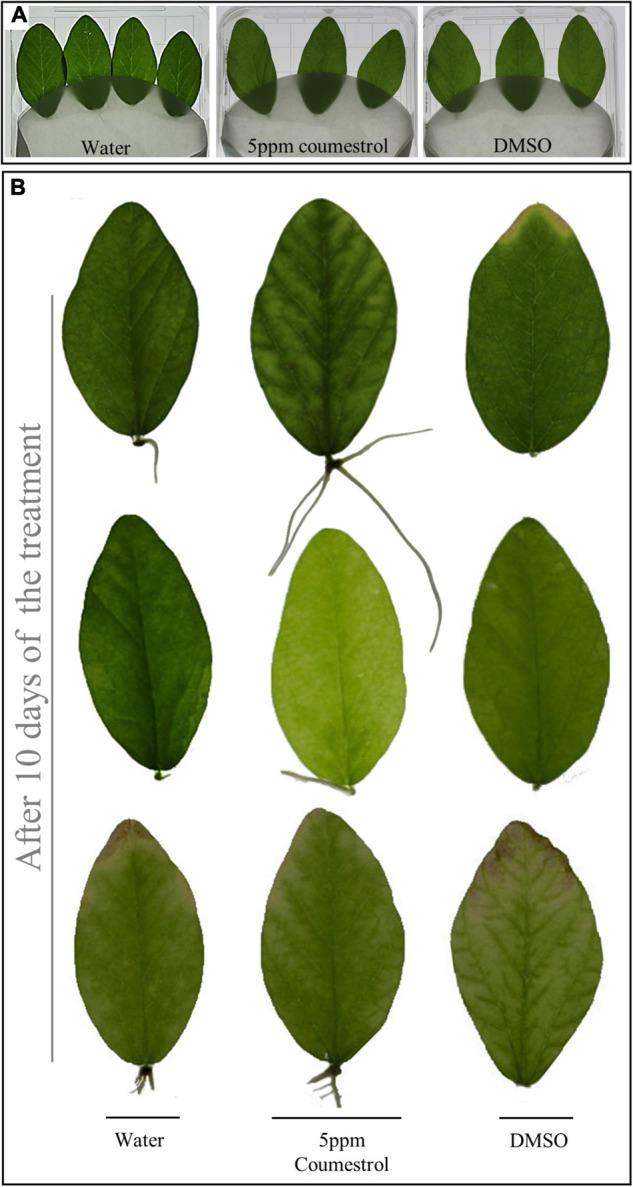
External application of coumestrol to soybean leaves. Leaves from soybean plants were treated with water, an optimized concentration of 5 ppm coumestrol, and dimethyl sulfoxide (DMSO) **(A)**. Replicated leaf images showing the development of roots after 10 days of the treatment with coumestrol only **(B)**.

According to the HPLC results shown in Figure 1, as aging in soybean plants progresses, the accumulation of three coumestans gradually increases. As described above, coumestrol was the most abundant among the three coumestan compounds at R7 stage, this indicates that coumestrol contributes to changes in leaf color and senescence besides several other metabolites that may be involved in leaf senescence. Additionally, we identified two intriguing proteins (corresponding to spots 5 and 13 in Figure 2) that showed a significantly high accumulation at R7 compared to V1. MALDI-TOF-MS analysis was used to identify the two proteins as 3,9 -dihydroxypterocarpan 6A-monooxygenase (CYP93A1) and isoflavone reductase homolog 2 (IFR2). Putative CYP93A1 protein is involved in the biosynthesis of the phytoalexin glyceollin, whereas IFR2 encodes an enzyme involved in the biosynthesis of glyceollins from daidzein (Graham et al., 1990; Oliver et al., 2003). Diadzein is either converted to coumestrol or the pterocarpan glyceollins. The protein identification results were obtained from Mascot database search using peptide mass fingerprint. Mascot search results with scores greater than 55 are significant at *P* < 0.05. We got a score of 56 for CYP31A1. However, the peptide sequence matches of the whole CYP93A1 sequence was 14% (Supplementary Figure 5). We then blasted these matched amino acids on NCBI and got hit on GLYMA_03G143700v4 (*G. max*) which is the soybean CYP93A1 (Supplementary Figures 6A,B). This confirmed that the protein in the spot is CYP93A1. Thus, results of Figure 2, led us to the conclusion that various coumestans, especially coumestrol, contributes to leaf maturity and yellowing in soybean along with several other metabolites that may be involved in leaf senescence. To confirm this, we externally applied coumestrol to V1-stage soybean leaves detached from the top of the plant (Supplementary Figure 2). Ten days after the application, the leaves showed dramatic phenotypic and physiological changes. After applying 5-ppm coumestrol, the leaves exhibited an interesting development of roots along with chlorosis or yellowing of the leaves (Figure 3). Similar results of root development were obtained when we applied coumestrol to mung bean leaves (unpublished). The development of roots from leaves is an unusual phenomenon and has not been studied in detail. Lozovaya et al. (2005) reported the accumulation of various isoflavones (including, diadzine, and genistin), and isoflavonoid phytoalexins, including coumestrol in hairy soybean roots following *Fusarium solani* f. sp. *glycines* infection. Additionally, Lee et al. (2012) reported the accumulation of coumestrol in root nodules. Moreover, recently, Xu et al. (2021) reported that the reduction of root nodulation and rhizobial infection upon exogenous ABA application is related to the reduction of isoflavonoid compounds, including coumestrol. As indicated in Figure 3B, the yellow color that appeared throughout the leaf caused by coumestrol application looked similar to the changes that appear in soybean leaves when naturally aged. This indicates that coumestrol may also regulate root development in soybean in addition to its role in senescence.

### Phytohormone Application Triggers an Increase in Coumestrol Levels in Soybean Leaves

**FIGURE 4:**
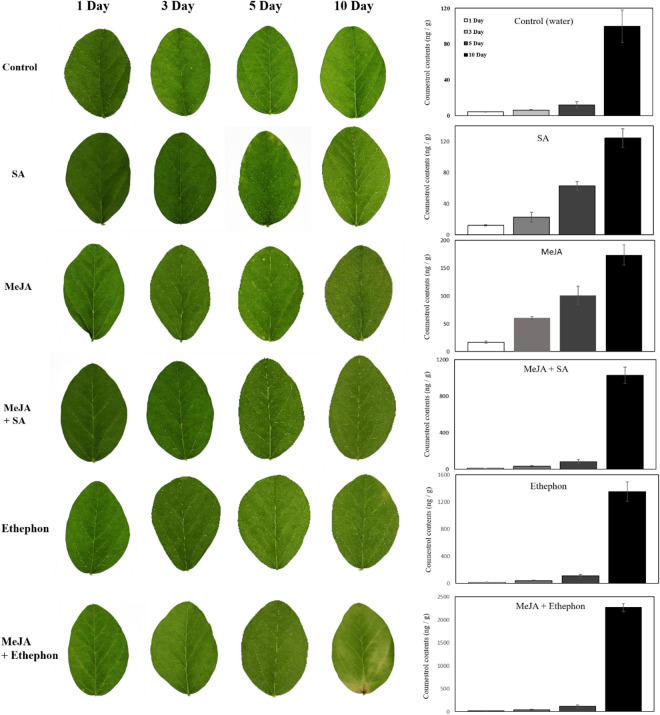
Effects of various phytohormones on soybean leaves and endogenous coumestrol content. Leaf chlorosis was observed after 5–10 days of phytohormone application and the graph at the right side shows concomitant increase in coumestrol contents after phytohormone application at different days.

Leaf senescence may be caused by various factors, such as the phytohormone, drought, and oxidative stress. Ethylene and cytokinin are well-known phytohormones connected with plant senescence. It was reported that coumestrol levels increase following insect attack to prevent plant damage (Loper, 1968). JA is a key hormone that helps plants protect themselves against insects or necrotrophic pathogens. There is a high possibility of the interaction between coumestrol and phytohormones. Thus, we investigated changes in coumestrol levels following the application of MeJA, SA, and ethylene. Following the application of these phytohormones, soybean leaves did not exhibit any significant phenotypic changes for up to 5 days (Figure 4). However, after 10 days of application, clear changes in leaf color was observed. The leaves treated separately with MeJA and SA turned light yellow with a concomitant increase in coumestrol content. Similar results were observed in leaves treated with MeJA and SA with an additive or synergistic effect, as indicated by the significant increase in coumestrol contents compared with water or MeJA and SA alone. Notably, ethephon (ethylene releasing compound) had an even stronger and highly significant effect than the other phytohormones. Leaves treated with ethephon alone or combined with MeJA started to turn yellow after 5 days of treatment and resulted in a significantly higher accumulation of coumestrol. Ethephon appeared to have the highest significant effect as the color of the leaves changed within 5 days of the application with more than ten times increase in the coumestrol content compared to the control plants. Ethephon and MeJA had an additive or synergistic effect on the coumestrol content after 10 days of treatment (Figure 4). Leaf chlorosis was observed after 5–10 days of phytohormone application. Leaves treated separately with MeJA and SA turned light yellow with a concomitant increase in coumestrol content. MeJA and SA, when combined, had an additive or synergistic effect on leaf color and coumestrol content as indicated by the significant increase in coumestrol contents compared to water, or MeJA and SA alone. The ethylene releasing compound, Ethephon, had an even stronger and highly significant effect than the other phytohormones. Leaves treated with ethephon alone or combined with MeJA started to turn yellow after 5 days of treatment and resulted in a significantly higher accumulation of coumestrol. Ethephon and MeJA had an additive or synergistic effect on the coumestrol content after 10 days of treatment.

### Coumestrol Biosynthetic Genes Are Affected by Exogenous Hormonal Application

**FIGURE 5:**
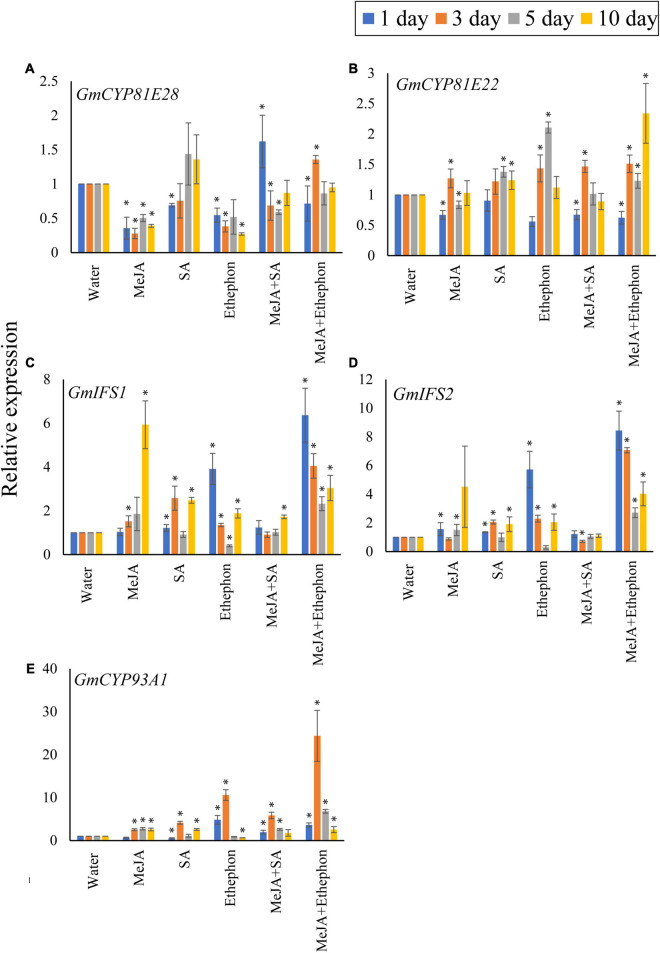
Real-time PCR analysis. Expression analysis of the coumestrol biosynthesis pathway genes. Five genes, **(A)** *GmCYP81E28*, **(B)** *GmCYP81E22*, **(C)** *GmIFS1*, **(D)** *GmIFS2*, **(E)** *GmCYP93A1* were checked. All data points represent the mean of three replicates. Error bars indicate standard error. Asterisks (*) indicate a significant difference between the means of the treatments and the corresponding control (water). Means with significant differences were separated using the Student’s *T*-test at 0.05% level of significance.

Next, we checked the expression levels of genes involved in coumestrol biosynthesis (Figure 5) following the application of various hormones alone or in combination as described above. Two genes, *CYP81E28* (Gm08G089500) and *CYP81E22* (Gm16G149300), are known to be involved in the hydroxylation of daidzein during coumestrol biosynthesis (Reinprecht et al., 2017). Overall, we did not find any statistical difference between the expression of *CYP81E28* in plants treated with any of the phytohormone alone or in combination after 3, 5, or 10 days. However, compared to the control plants treated with water only, a significant increase in expression was recorded after 5 and 10 days of SA application and 3 days of MeJA + Ethephon (Figure 5A). Generally, the phytohormones increased the expression of *CYP81E22* after 3, 5, and 10 days of phytohormone application compared to the control plants treated with water only (Figure 5B). More specifically, no significant difference was observed in the expression of *CYP81E22* after 3, 5, or 10 days of SA application. Ethephon treatment significantly increased the expression of *CYP81E22* after 5 days whereas, a significantly higher expression was recorded when it was applied along with MeJA (Figure 5B). The genes *GmIFS1* and *GmIFS2* function upstream of coumestrol biosynthesis and are involved in converting liquiritigenin to daidzein (Sohn et al., 2021). When compared to the control plants, we found a significant increase in the expression of *GmIFS1* after phytohormone application. MeJA induced a significantly high expression of *GmIFS1* after 10 days of application (Figure 5C). However, changes in the expression of *GmIFS2* were largely non-significant following phytohormone treatment. However, the highest significant expression was recorded after 3 days of the combined treatment of MeJA and ethephon (Figure 5D). The highest change in expression after phytohormone treatment was recorded for *GmCYP93A1*, which is involved in the final step of coumestrol biosynthesis. All the phytohormones significantly increased the expression compared to the control plants treated with water only. A significant increase in expression after 3, 5, and 10 days was recorded following MeJA treatment. The highest *GmCYP93A1* expression was recorded after 3 days of ethephon treatment, which increased by twofolds when ethephon was applied along with MeJA (Figure 5E).

## Discussion

**FIGURE 6:**
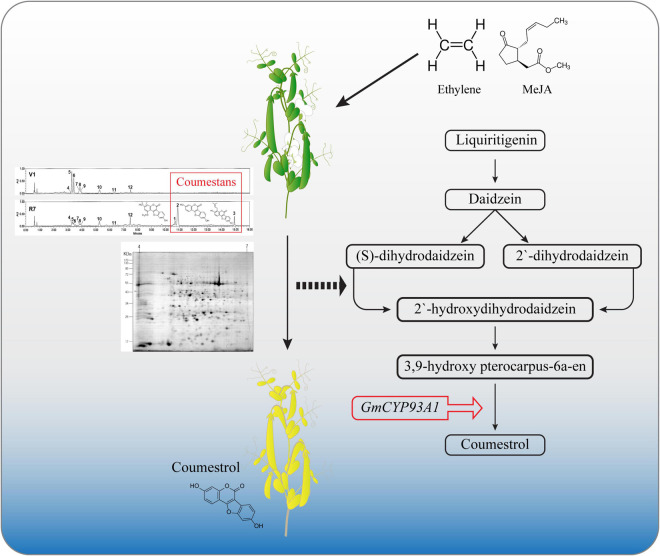
Schematic diagram showing the role of coumestans in soybean senescence. Significantly higher accumulation proteins involved in regulating secondary metabolites, such as glutamine synthetase, 3,9 -dihydroxypterocarpan-6A-monooxygenase, and isoflavone reductase homolog 2 were detected in mature soybean leaves (R7 stage). The 3,9 -dihydroxypterocarpan 6A-monooxygenase encoded by *CYP93A1*, is an enzyme involved in the final step of coumestrol biosynthesis. The application of phytohormones alone and in different combinations significantly increased the expression of *CYP93A1*. External application of 5 ppm coumestrol induced senescence in soybean leaves characterized by yellowing of the leaves.

Soybean is one of the main crops consumed by humans because of its nutritional value. Recently, secondary metabolites of soybean, such as flavonoids and phenolics, have been studied to a great extent due to their beneficial effects on human health. Yuk et al. (2011b) described the α-glucosidase inhibition activity of soybean leaf extracts. They found that coumestrol was the most abundant polyphenol, indicating the importance of coumestrol and other coumestans in the armada of secondary soybean metabolites. This study further highlights the role of coumestans, especially coumestrol in soybean senescence. The results of this study, were summarized diagrammatically in Figure 6. A diverse array of secondary metabolites is produced in soybean leaves. The production of secondary metabolites in plants is influenced by environmental conditions. Various phytohormonal signaling pathways regulate plant responses to environmental changes (Wani et al., 2016). Leaf mutuality or senescence is tightly linked to phytohormones, such as ethylene, jasmonates, SA, and cytokinins. Thus, this research aimed to investigate the correlation between senescence and coumestrol content, and their relationship with various phytohormones. Our initial investigations describe the significance of three coumestans in soybean senescence. These different coumestans are isotrifoliol, coumestrol, and phaseol and were detected only in mature leaves at the R7 growth stage. This was in sharp contrast to at least eight other chemical compounds, including flavonoid glycosides, detected only in the fresh V1-stage leaves (Figure 1). Groenbaek et al. (2019) reported a gradual but significant decrease in the flavonoid glycoside groups as plants age. They function to protect the young leaves from solar radiation damage with no significant contribution in leaf color changes in rapeseed (*Brassica napus*). However, glycosyloxy-isoflavone groups, such as daidzein, and genistin quantities increase during senescence (Groenbaek et al., 2019). These compounds function in the same pathway for the production of leguminous isoflavonoids (Shimamura et al., 2007). Next, this study describes the involvement of two proteinsin the regulation of secondary metabolites. These two proteins encode the enzymes; glutamine synthetase, 3,9 -dihydroxypterocarpan 6A-monooxygenase, and isoflavone reductase homolog 2. Interestingly, these proteins were significantly more abundant in mature leaves than young leaves, highlighting the variations in leaf chemistry during senescence. The 3,9 -dihydroxypterocarpan 6A-monooxygenase encoded by *CYP93A1* is an enzyme involved in the final step of coumestrol biosynthesis. The application of phytohormones significantly increased the transcript accumulation of *CYP93A1* in soybean leaves with a concomitant yellowing of leaves. This indicates a key role of coumestrol in transcriptional programming, phytohormonal regulation, and changes in leaf chemistry during senescence. However, it is important to mention that the molecular weight of spot 5 CYP93A1 did not match well with its theoretical Pi/MW. The molecular weight was 17 kDa. Whereas the theoretical weight is around 57 kDa. Interestingly, the molecular weight was close to the theoretical MW weight of SPOT 15 which was found as an unknown protein and it may possibly be partial CYP93A1 protein. On the other hand, the Pi and MW of IFR2 protein at (Spot 13) matched well with its theoretical Pi and MW (theoretical pI/Mw = 5.60/33,939.58). The role of coumestrol in leaf senescence was further confirmed by exogenous application of coumestrol to fresh soybean leaves that turned yellow (just like naturally mature/senescent leaves) after application of just 5-ppm coumestrol, which interestingly is itself a yellow-colored chemical. The surprising development of roots from the base of the petiole after the application of coumestrol to the leaves was unexpected and unclear. However, it indicates the involvement of coumestrol in root induction, though it needs further investigations. The involvement of coumestrol in root development and other root-related phenomena has been reported earlier. Lozovaya et al. (2004) reported the accumulation of various isoflavones (including diadzine, and genistin), and isoflavonoid phytoalexins, including coumestrol in soybean hairy roots following *F. solani* f. sp. *glycines* infection, indicating its role in disease response. Additionally, Lee et al. (2012) reported the accumulation of coumestrol in root nodules. Moreover, recently Xu et al. (2021) reported the reduction of root nodulation and rhizobial infection upon exogenous. Future investigations into the role of coumestrol in senescence, root development, and disease resistance can provide new insights into the roles of this important secondary metabolite.

## Data Availability Statement

The datasets presented in this study are included in the article/Supplementary Material, further inquiries can be directed to the corresponding author (B-WY, bwyun@knu.ac.kr).

## Author Contributions

HY and B-GM designed the experiments. H-HK and B-GM conducted the experiments and wrote the manuscript. HY, H-HK, and B-GM conducted data analysis. AH, GL, and B-WY undertook the critical review and editing. AH, B-GM, and GL revised the manuscript. B-WY provided the supervision. All authors have read and agreed to the published version of the manuscript.

## Conflict of Interest

The authors declare that the research was conducted in the absence of any commercial or financial relationships that could be construed as a potential conflict of interest.

## Publisher’s Note

All claims expressed in this article are solely those of the authors and do not necessarily represent those of their affiliated organizations, or those of the publisher, the editors and the reviewers. Any product that may be evaluated in this article, or claim that may be made by its manufacturer, is not guaranteed or endorsed by the publisher.

## Footnotes

1
https://prospector.ucsf.edu/prospector/mshome.htm

## Supplementary Material

Supplementary Figure 1 Gradual changes in the color of soybean leaves at the different growth stages. Soybean leaves begin to lose their green color from the R2 stage onward. Leaves turn completely yellow at the mature or senescent R7 stage **(A)**. The loss of green color or senescence is accompanied by a concomitant loss of the total chlorophyll content. A significant increase in the chlorophyl content was recorded up to V5 which then starts to reduce, with the least chlorophyl content at the R7 stage **(B)**.

Supplementary Figure 2 Identification of the compound 1 (Isotrifoliol) carried out based on fragmentation pattern of MS/MS. UV scanning (200–400 nm) spectra **(A)**. Positive ion mode was acquired for mass fragmentation spectra **(B)**. Elemental composition analysis using HR-ESI/MS **(C)**.

Supplementary Figure 3 Identification of the compound 1 (Coumestrol) carried out based on fragmentation pattern of MS/MS. UV scanning (200–400 nm) spectra **(A)**. Positive ion mode was acquired for mass fragmentation spectra **(B)**. Elemental composition analysis using HR-ESI/MS **(C)**.

Supplementary Figure 4 Identification of the compound 1 (Phaseol) carried out based on fragmentation pattern of MS/MS. UV scanning (200–400 nm) spectra **(A)**. Positive ion mode was acquired for mass fragmentation spectra **(B)**. Elemental composition analysis using HR-ESI/MS **(C)**.

Supplementary Table 1 Identified protein list by MALDI-TOF-MS.

Supplementary Table 2 List of primers.

## Abbreviations

DMSO: dimethyl sulfoxide
MeJA: methyl jasmonate
ET: etheylene
SA: salicylic acid
HPLC: high-performance liquid chromatography
SDS-PAGE: S sodium dodecyl sulfate poly acrylamide gel electrophoresis
2-DE: 2-dimensional gel electrophoresis.
