# Supplementary figures and images for: A Potential Role of Coumestrol in Soybean Leaf Senescence and Its Interaction With Phytohormones
Source: Front Plant Sci. 2021 Nov 25;12:756308. doi: 10.3389/fpls.2021.756308 (PMC8655741; doi:10.3389/fpls.2021.756308)

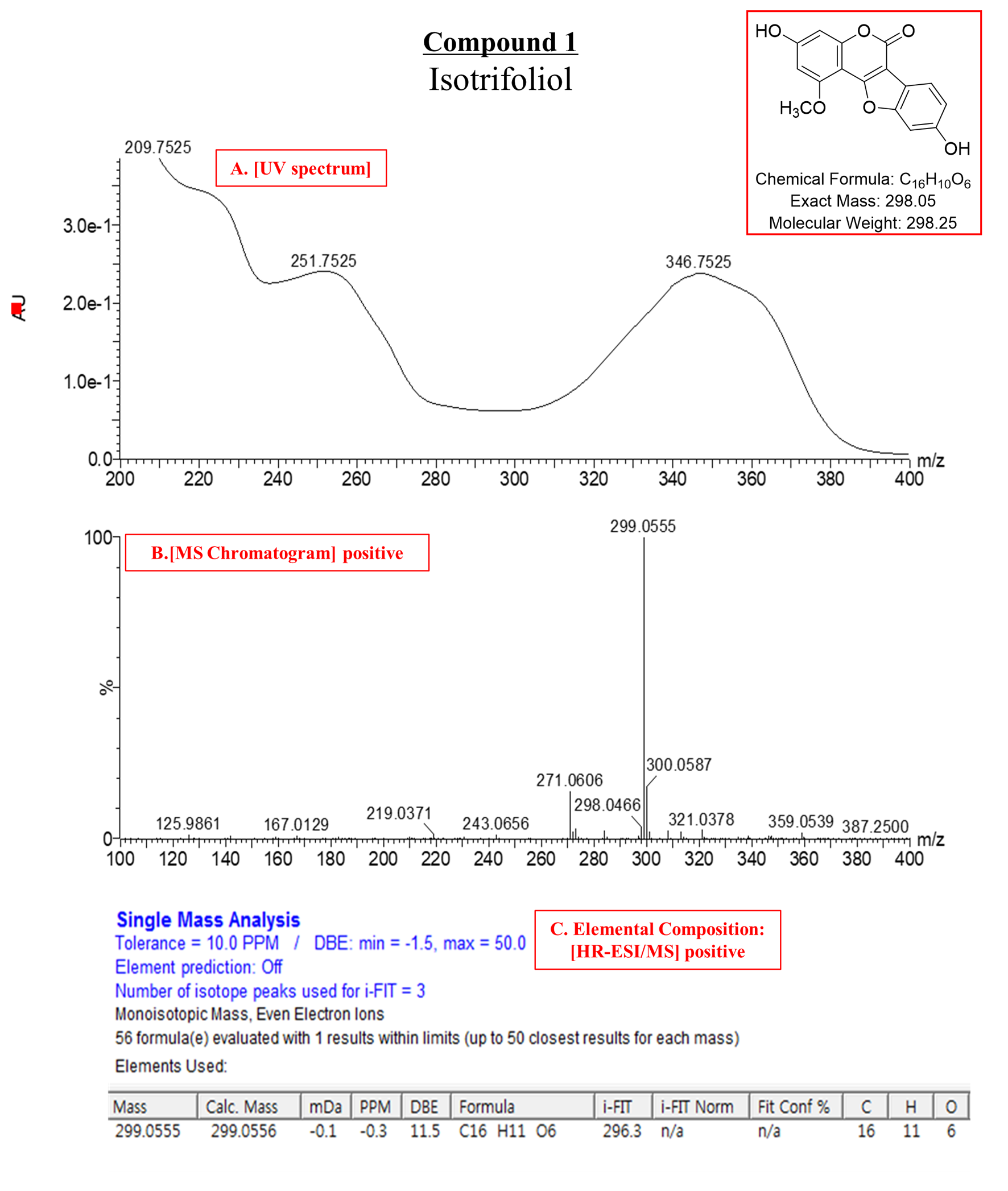

Supplement: Supplementary Figure 1 — Gradual changes in the color of soybean leaves at the different growth stages. Soybean leaves begin to lose their green color from the R2 stage onward. Leaves turn completely yellow at the mature or senescent R7 stage (A). The loss of green color or senescence is accompanied by a concomitant loss of the total chlorophyll content. A significant increase in the chlorophyl content was recorded up to V5 which then starts to reduce, with the least chlorophyl content at the R7 stage (B). [file Image_1.TIF]

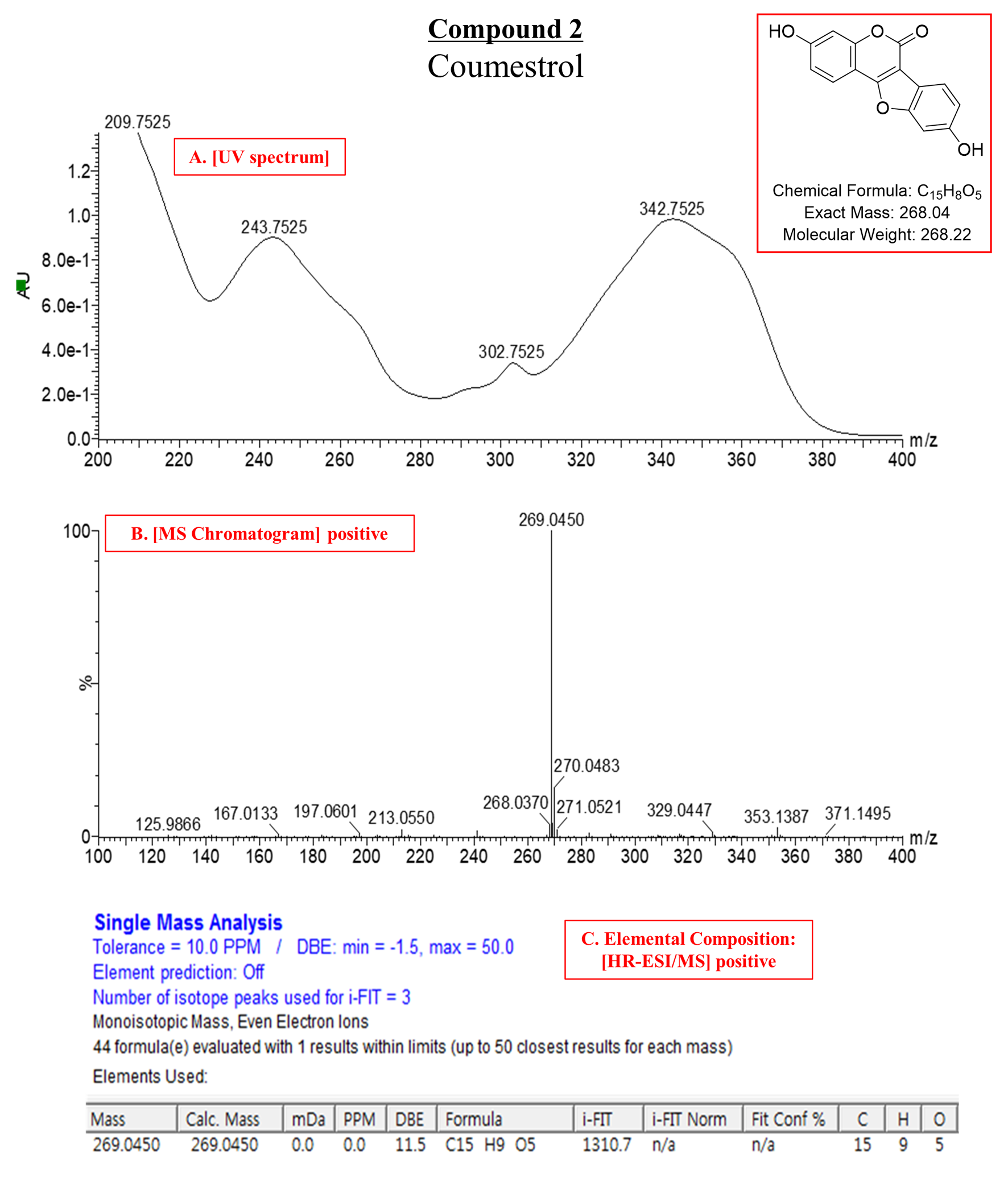

Supplement: Supplementary Figure 2 — Identification of the compound 1 (Isotrifoliol) carried out based on fragmentation pattern of MS/MS. UV scanning (200–400 nm) spectra (A). Positive ion mode was acquired for mass fragmentation spectra (B). Elemental composition analysis using HR-ESI/MS (C). [file Image_2.TIF]

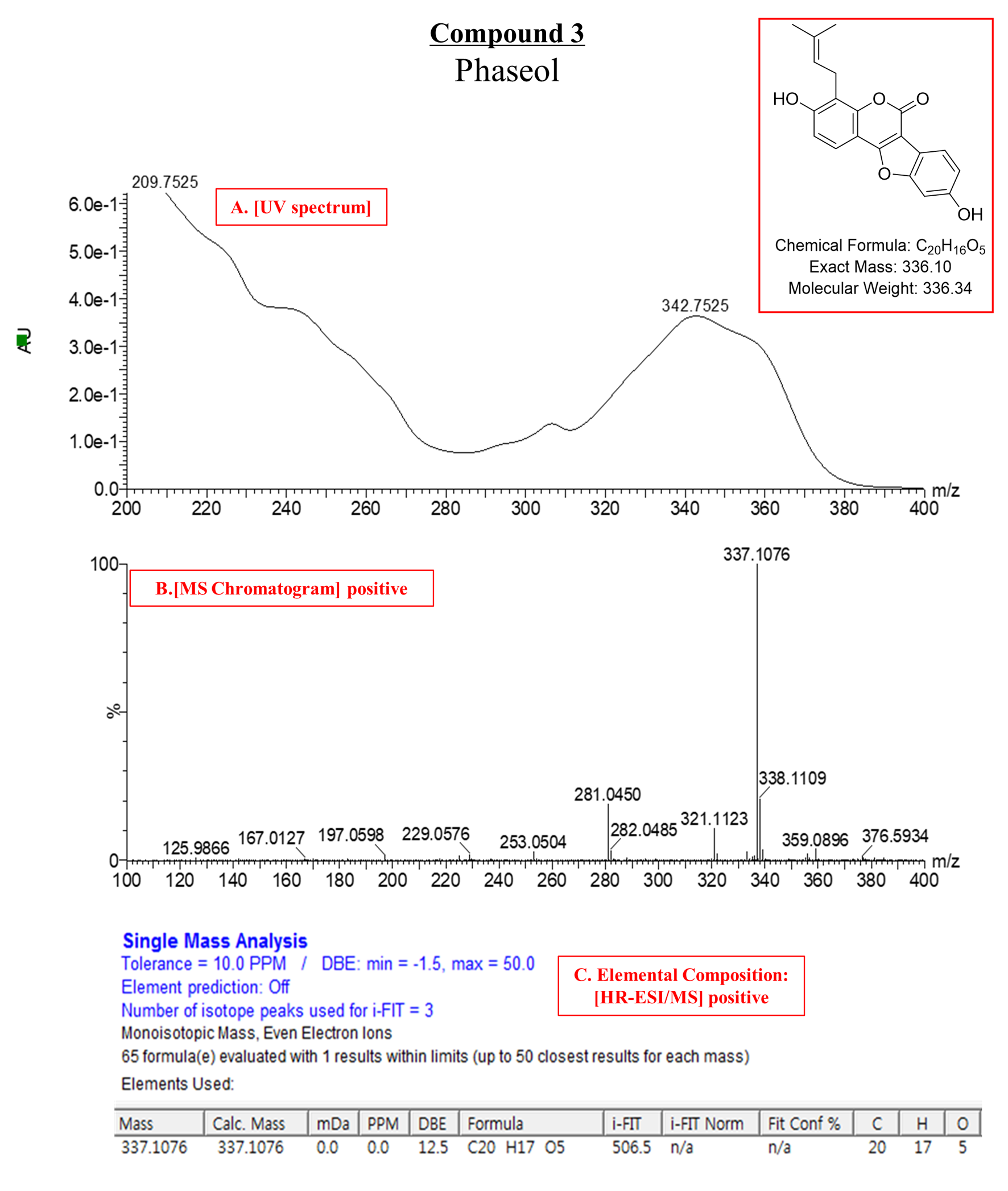

Supplement: Supplementary Figure 3 — Identification of the compound 1 (Coumestrol) carried out based on fragmentation pattern of MS/MS. UV scanning (200–400 nm) spectra (A). Positive ion mode was acquired for mass fragmentation spectra (B). Elemental composition analysis using HR-ESI/MS (C). [file Image_3.TIF]

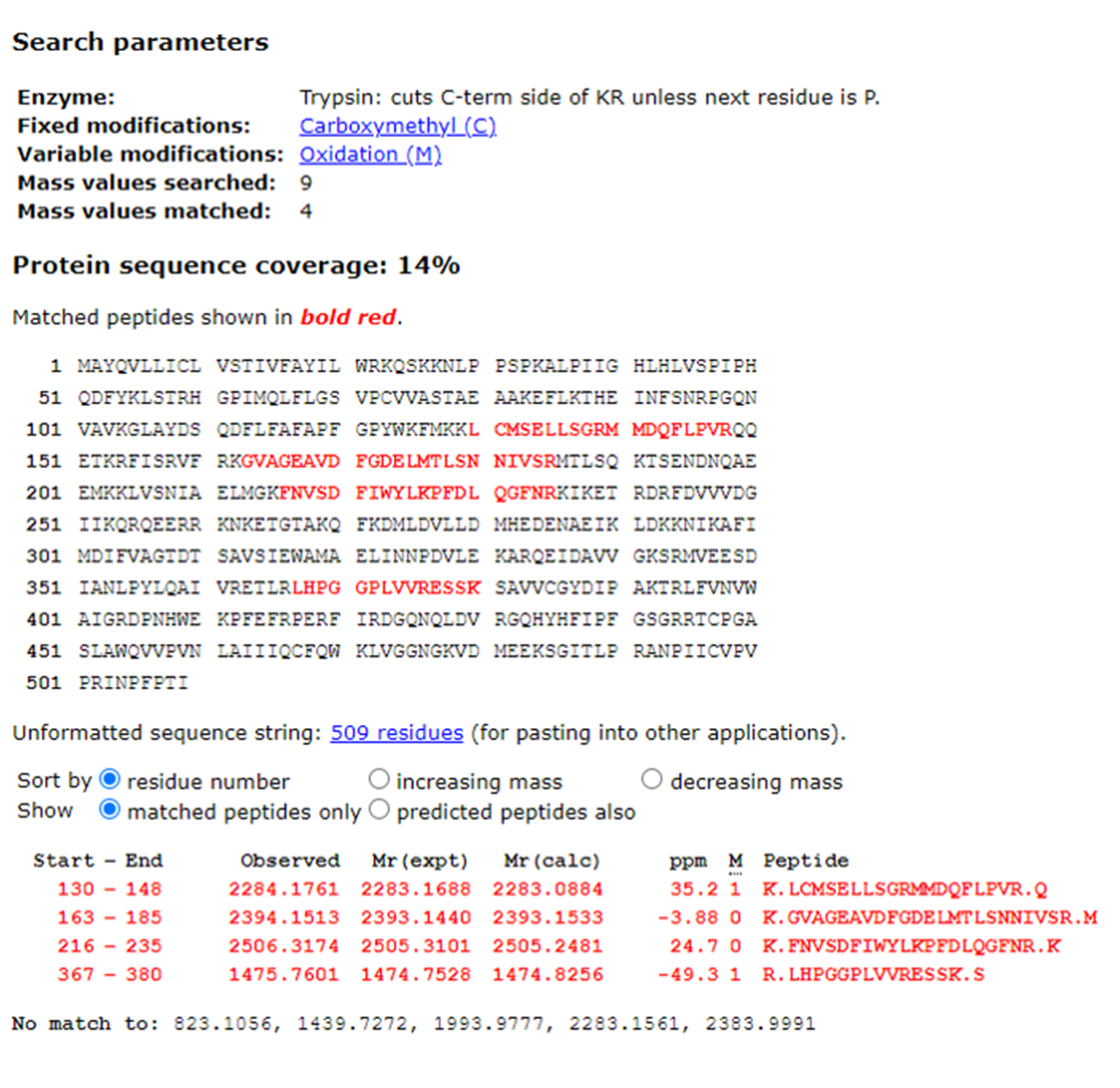

Supplement: Supplementary Figure 4 — Identification of the compound 1 (Phaseol) carried out based on fragmentation pattern of MS/MS. UV scanning (200–400 nm) spectra (A). Positive ion mode was acquired for mass fragmentation spectra (B). Elemental composition analysis using HR-ESI/MS (C). [file Image_4.TIF]

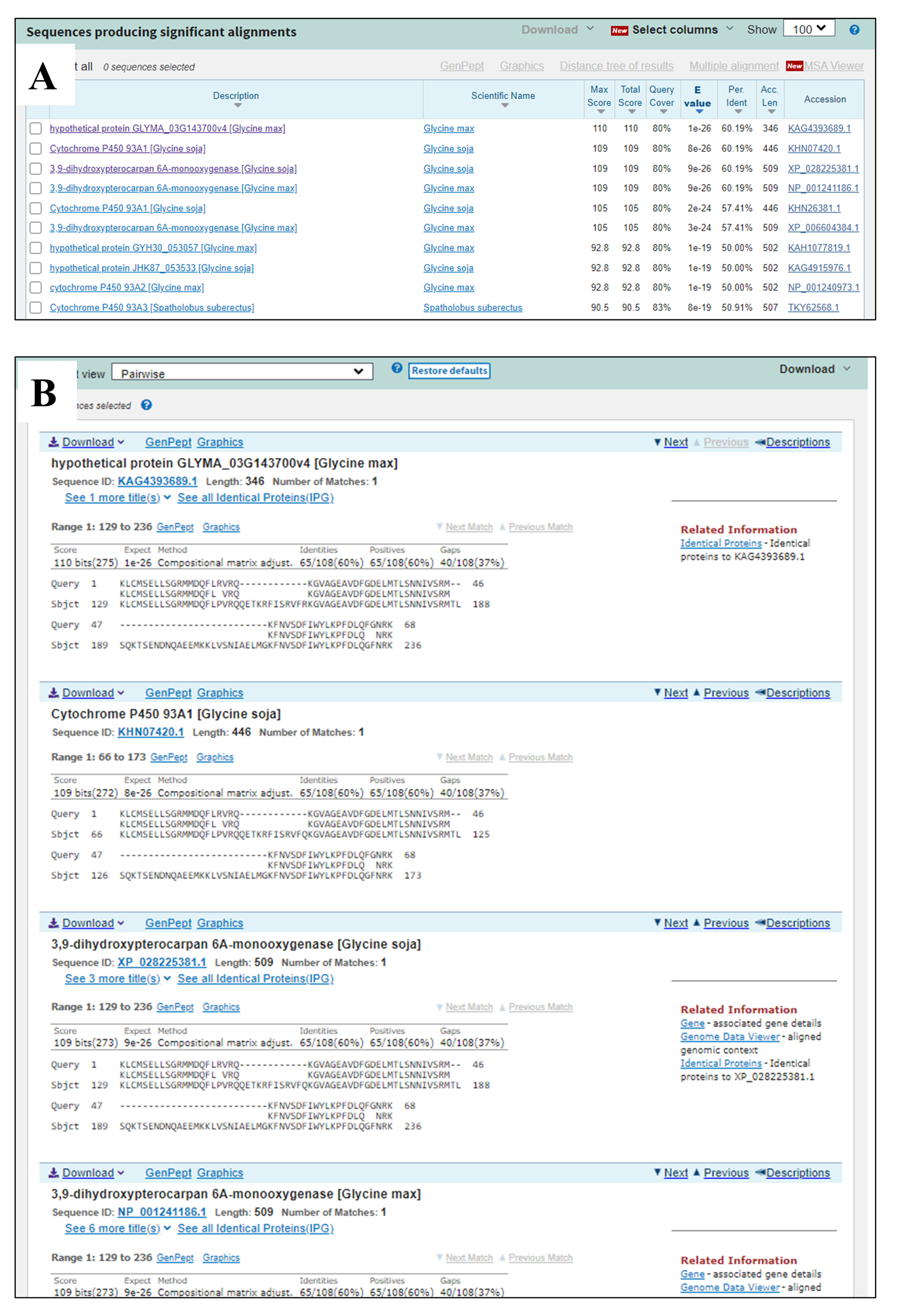

Supplement: Supplementary file 7 [file Image_5.TIF]

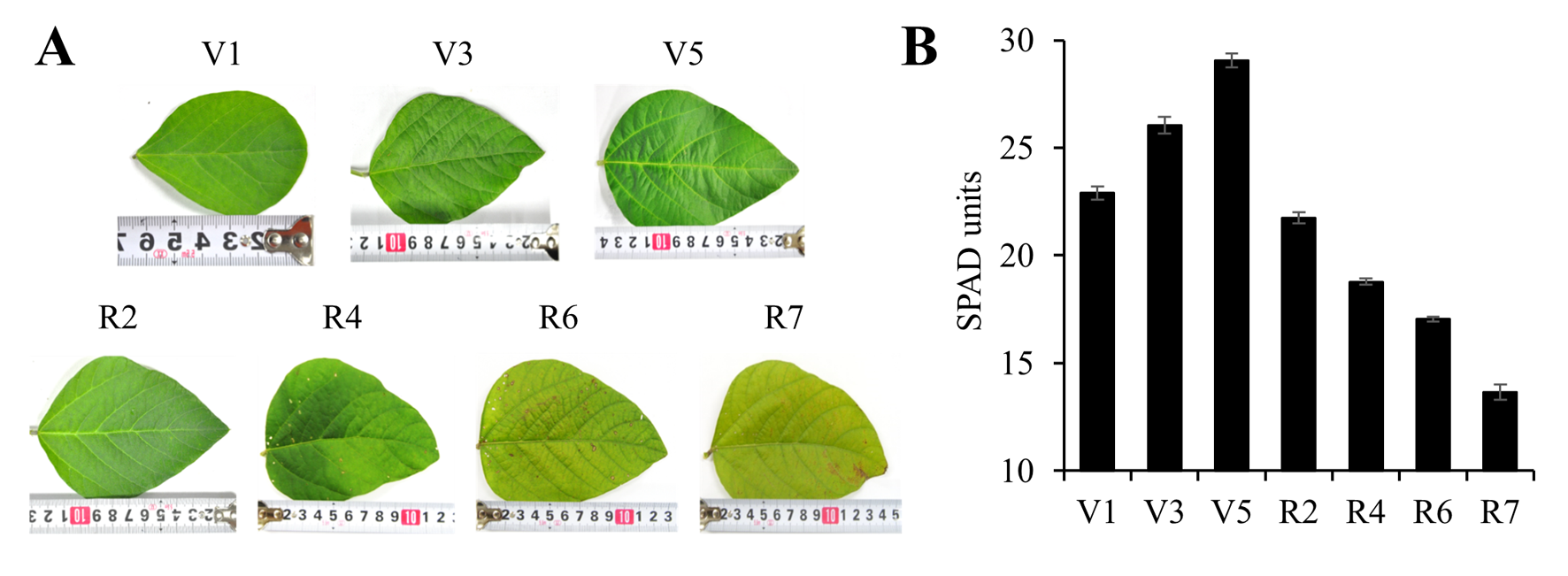

Supplement: Supplementary file 8 [file Image_6.TIF]
